# The Role of Storage Lipids in the Relation between Fecundity, Locomotor Activity, and Lifespan of *Drosophila melanogaster* Longevity-Selected and Control Lines

**DOI:** 10.1371/journal.pone.0130334

**Published:** 2015-06-26

**Authors:** Neda Nasiri Moghadam, Martin Holmstrup, Tommaso Manenti, Marie Brandt Mouridsen, Cino Pertoldi, Volker Loeschcke

**Affiliations:** 1 Department of Bioscience, Aarhus University, Ny Munkegade 114, DK-8000 Aarhus C, Denmark; 2 Department of Bioscience, Aarhus University, Vejlsøvej 25, PO Box 314, DK-8600 Silkeborg, Denmark; 3 Aalborg Zoo, Mølleparkvej 63, 9000 Aalborg, Denmark; 4 Department 18 / Section of Environmental Engineering, Aalborg University, Sohngårdsholmvej 57, 9000 Aalborg, Denmark; Virginia Commonwealth University, UNITED STATES

## Abstract

The contribution of insect fat body to multiple processes, such as development, metamorphosis, activity, and reproduction results in trade-offs between life history traits. In the present study, age-induced modulation of storage lipid composition in *Drosophila melanogaster* longevity-selected (L) and non-selected control (C) lines was studied and the correlation between total body fat mass and lifespan assessed. The trade-offs between fecundity, locomotor activity, and lifespan were re-evaluated from a lipid-related metabolic perspective. Fewer storage lipids in the L lines compared to the C lines supports the impact of body fat mass on extended lifespan. The higher rate of fecundity and locomotor activity in the L lines may increase the lipid metabolism and enhance the lipolysis of storage lipids, reducing fat reserves. The correlation between neutral lipid fatty acids and fecundity, as well as locomotor activity, varied across age groups and between the L and C lines. The fatty acids that correlated with egg production were different from the fatty acids that correlated with locomotor activity. The present study suggests that fecundity and locomotor activity may positively affect the lifespan of *D*. *melanogaster* through the inhibition of fat accumulation.

## Introduction

Aging strongly provokes fat accumulation via reduced insulin sensitivity [1] and inhibition of lipolysis [2], affecting lifespan. Clancy et al. [3] demonstrated that mutation of the gene *chico*, which encodes an insulin receptor substrate in *Drosophila melanogaster*, extends the median and maximum lifespan of females by 48% and 41%, respectively. In addition, over-expression of downstream target of insulin-like growth factor (dFOXO protein) in the head fat body of adult *Drosophila* has been shown to alter lipid metabolism and increase the lifespan of both males and females [4].

Evidence from the last few years supports the hypothesis of a negative correlation between the body fat mass and lifespan. The link between storage lipids and lifespan arises from the contribution of fat-mass-derived signaling factors to the maintenance of insulin sensitivity, lipid metabolism, and energy homeostasis [5]. Reduced fat mass eliminates the risk of aging-associated phenotypes, such as cardiovascular disease, type 2 diabetes, and some types of cancer [5–7]. Among fat-mass-derived signaling factors, adipokinetic hormone has a considerable influence in slowing or preventing age-related diseases through stimulating lipid metabolism or reducing the synthesis of lipids [8,9].

The link between body fat mass and longevity is not without controversy. In a microRNA study by Zhu et al. [10], the fat content of *Caenorhabditis elegans lin4*-mutants was considerably lower and their lifespan shorter than that of wild-type individuals. In *D*. *melanogaster*, for a given caloric intake, manipulation of dietary components (sugar- or yeast-enriched diet) produces very lean (protein-rich diets) or obese (sugar-rich diets) flies with a short lifespan [11]. However, in most previous studies, lifespan extension via caloric/food restriction or variation in nutritional components was always accompanied by a reduction in total body fat mass [12–14].

The lower amount of storage lipids in food-restricted animals is a consequence of enhanced insulin sensitivity and improved lipolysis of storage lipids [9,15]. Because fat lipolysis relies on specific fat body lipases, lifespan extension through dietary restriction may be mediated by an increase in lipase activity. In *C*. *elegans*, expression of a triglyceride lipase (*lipl-4*) in germline-ablated individuals eliminated lipid accumulation in the fat cells and extended lifespan [7]. These responses upon germline arrest suggest that lipolysis controls the process of aging through the regulation of lipid homeostasis.

Another effective factor on the rate of aging is the molecular configuration of fatty acids that contribute to the composition of storage lipids. As endogenous fuels in organisms, fatty acids undergo various metabolic pathways in accordance with their structural properties such as acyl chain length and the degree of unsaturation. These features affect molecule size, energy content, and the rate of digestion, absorption, and mobility of fatty acids to utilization sites [16]. For example, the intestinal hydrolysis and absorption of medium chain fatty acids (8–12 C atoms) is faster and more efficient than that of equivalent long chain fatty acids (14 or more C atoms) because of their low molecular weight [16].

Insects store neutral lipid fatty acids (NLFAs), mainly in the form of triglyceride in lipid droplets. These small storage compartments are the basic constitutive units of the fat body [17], comprising a considerable percentage of the insect’s fresh weight with heterogeneous distribution throughout the body. The insect fat body provides a metabolic center to satisfy the energy requirements of manifold physiological and behavioral aspects, such as metamorphosis, diapause, embryogenesis, and flight [18,19]. Various factors, including nutrition, age, sex, and development stage affect the energy content of the lipid droplets via altered fatty acid composition [20–22].

When energy is required, neutral lipids undergo lipolytic degradation by lipases and hydrolases, releasing fatty acids as energy substrates into the hemolymph. As the major circulatory system of insects, hemolymph is in close contact with lipid droplets. This feature facilitates the efficient transfer of energy substrates from the fat body to utilization sites, such as flight muscles [17]. The process of lipid hydrolysis is regulated in part by complex hormonal signals and catalyzed by fat body triacylglycerol lipases. Therefore, these two factors play a fundamental role in maintaining lipid homeostasis.

Simultaneous utilization of the fat body as a major source of energy by multiple processes in insects results in inevitable complex trade-offs between costly traits; an increase in a life history trait is usually accompanied by a reduction in another trait. This feature modulates the allocation of energy to multiple life history traits and maintains energy homeostasis, which is essential for the survival and reproductive success of an organism [23]. Such trade-offs have been observed between egg production, locomotor activity, and lifespan in a pair-wise manner in previous studies using model organisms; individuals with reduced fecundity or activity lived longer than those with a high rate of fecundity or activity [24–26]. However, a longer lifespan is not always accompanied by lower fecundity or locomotor activity [27,28]. For example, in a study by Simon et al. [29], mutation of the ecdysone receptor gene (*EcR*) in *D*. *melanogaster* increased the average lifespan of heterozygous offspring without any reduction in fecundity or activity. Furthermore, the negative relationship between longevity and reproduction in response to dietary restriction uncoupled in *Drosophila* after supplementing the medium with methionine [12]. These contradictory observations reflect the complexity of correlations among life history traits.

According to recent studies, body fat mass may be the causal factor by which fecundity, locomotor activity, and lifespan are linked [30–32], though the importance of fat content and its composition to the interconnection between these traits has remained obscure. The present study aimed to determine the age-induced variation in fat storage composition and its role in fecundity, locomotor activity, and lifespan in *D*. *melanogaster* longevity-selected (L) and non-selected control (C) lines. The recently proposed hypothesis of a negative correlation between total body fat mass and lifespan was also evaluated [6].

## Materials and Methods

### Stock population

The three replicate L lines and corresponding C lines of *D*. *melanogaster* used in this study were derived from the fourth generation of a mass bred population established in 2002 by mixing flies from different geographic regions [33]. The L lines were selected to postpone senescence via gradual extension of generation time so that the median lifespan of the L mated females after 31 generations of selection was 66% longer on average than the lifespan of C individuals [34].

### Experimental set-up

For the purposes of the present study, the lines were cultured in 300 mL plastic bottles (5 bottles per replicate, approximately 150 flies per bottle) containing 70 mL of standard *Drosophila* oatmeal-agar-sugar-yeast medium (0.03, 0.01, 0.04, and 0.06 g/mL, respectively) and maintained in a common environment (25°C and 12:12 light:dark cycle) for two generations to avoid maternal effects. The presence of fatty acids in the medium was assessed for six samples (each one containing 5 mg medium) of the standard *Drosophila* medium. The samples were placed separately in Eppendorf tubes for extraction using the same method as for storage lipids (see the storage lipid characterization section). Due to the presence of oatmeal and yeast (both of these ingredients have a natural content of lipids) in the medium, the standard *Drosophila* diet contained the following fatty acids: myristic (C_14:0,_ 0.05 mg/g medium), palmitic (C_16:0_, 0.11 mg/g medium), palmitoleic (C_16:1,_ 0.07 mg/g medium), stearic (C_18:0_, 0.02 mg/g medium), oleic (C_18:1,_ 0.18 mg/g medium), and linoleic acid (C_18:2,_ 0.19 mg/g medium). At 3 days of age, eclosed flies from the second generation were allowed over a 3-hour window to oviposit on small spoons filled with ~1 mL standard *Drosophila* medium seeded with live yeast. Eggs were collected at controlled densities (40 eggs per vial) and placed into 35 mL plastic vials (50 vials per replicate) containing 7 mL standard *Drosophila* medium. Flies were collected within 24 hours of the first eclosion. Generally, collected flies were aged for one day before separation under CO_2_ anesthesia, and each set of 40 female flies was kept in a 35 mL plastic vial containing 3 mL of standard *Drosophila* medium. Given the short virgin period (time between eclosion and first mating) of the females, which was <16 hours on average in both selection regimes [28], the presence of virgin females at the time of sexing was unlikely. However, to ensure that all females were mated three males were placed in each vial. The stock population and experimental flies were maintained at 25°C in a climate-controlled room under a 12:12 light:dark cycle. All phenotypic assays were performed under these same conditions.

### Fitness traits

#### Fecundity

The fecundity assay was started at 3 days of age and continued for 17 days by placing 30 mated females from each replicate (180 flies in total) individually into empty 35 mL plastic vials containing a small spoon filled with ~1 mL of standard *Drosophila* medium. The spoons were replaced every day at 10 a.m. and the eggs counted manually immediately afterwards under a dissecting microscope. Data from flies that died during the experiment (8 out of 180 individuals) were excluded from the analyses.

#### Locomotor activity

The locomotor activity of mated female flies was recorded at 3, 9, 14, and 19 days of age based on a non-repeated measurement design using *Drosophila* activity monitors (DAMs) in which infrared detectors scored the activity when a fly crossed the beam [35]. A previous study of the lines used for the present experiment showed a high mortality rate of non-selected flies around 20 days of age [28]. Therefore, the assay was stopped at 19 days of age to avoid the effect of mortality on the activity assessment. At each specific age, samples (30 flies per replicate) were individually placed in a narrow glass tube (5 mm × 65 mm) with ~3 mm of *Drosophila* medium at one end that was closed with parafilm to last for ~48 hours, and with a cotton stopper at the other end for air ventilation. The glass tubes were placed in the DAMs randomly, with each monitor containing five samples of each replicate, and the activity was recorded every 15 seconds. At each specific age, the monitoring started at 1 p.m. and lasted for 26 hours. The sum of the activity counts recorded within a 24-hour window (the first and last hours of recorded data were excluded) was calculated as the total activity for an individual. The data for flies that died or escaped during the experiment (6 out of 720 individuals) were excluded from the analyses.

#### Storage lipid characterization

To assess the impact of age and longevity selection on the storage lipid profile of *D*. *melanogaster*, flies were collected within 24 hours of eclosion and kept at a controlled density in 35 mL plastic vials (40 flies per vial) containing 3 mL of standard *Drosophila* medium. During maintenance, the flies were transferred to new vials with fresh food every other day. At 3, 9, 14, and 19 days of age, samples were snap frozen in liquid N_2_ at the same time of day (2 p.m.) and sorted into sexes in a cooling room (4°C) to reduce the effect of ambient temperature on fatty acid composition. Batches containing 10 female flies were preserved at -80°C for subsequent analyses.

Prior to lipid extraction, samples were freeze-dried for 24 hours and weighed using a Sartorius MC5 microanalytical balance with an accuracy of ±1μg. For the randomized block experimental design, samples were assigned to three blocks, each containing 24 samples (2 selection regimes × 3 replicates × 4 age groups). NLFAs were extracted from intact flies using a modified Bligh-Dyer single-phase method [36] with 2:1:1 v/v/v chloroform:methanol:phosphate buffer in two steps [37]. Samples were centrifuged and the chloroform phase transferred to test tubes and evaporated to dryness under a stream of N_2_. The crude lipid extract was re-dissolved in a small volume of chloroform and added to pre-packed silica columns (100 mg silicic acid; Isolute, Mid Glamorgan, UK) placed on a vacuum manifold. NLFAs were eluted with chloroform, collected in test tubes, and dried under a stream of N_2_ before trans-methylation using the method of Dowling et al. [38]. Single chain fatty acid methyl esters (FAMEs) were then dissolved in heptane for further analyses (see [39] for more details).

FAMEs were identified by GC-MS with the initial temperature set at 50°C, initial time of 2 min, ramp of 20°C min^-1^, final temperature of 240°C, final time of 5 min, split ratio of 1:10, injection volume of 1 μL and injector temperature of 220°C [39]. NLFAs were identified by a comparison of the obtained mass spectra and known FAME standards (Nu-check Prep, Inc., Elysian, MN, USA). The mass concentration of individual fatty acids was calculated based on the area under the identified peaks with GC-MS Solution software, and the amount of each fatty acid (mg/mg dry weight) was calculated as a percentage of TFAMEs. Fatty acids were represented by their common names and designated as *x*:*y* (ώn), where *x* is the number of carbon atoms, *y* the number of double bonds and n the number of the first double bonded carbon atom counting from the methyl end (ώ). Using the proportion (on a mass basis) of fatty acids present in the NLFA extract, (un)saturation indices were calculated as follows: total amount of FAMEs (TFAMEs), sum of saturated fatty acids (ΣSFAs), sum of monounsaturated fatty acids (ΣMUFAs), the ratio of unsaturated (MUFAs and polyunsaturated fatty acid = PUFA) to saturated fatty acids (U/S) = (ΣMUFAs+PUFA)/ΣSFAs, average chain length (ACL) = [(%C_12_ × 12) + (Σ%C_14_ × 14) + (Σ%C_16_ × 16) + (Σ%C_18_ × 18)]/100 and peroxidation index (PI) = (ΣMUFAs × 0.025) + (PUFA × 1).

### Statistical analysis

General linear models were used with JMP version 10 using selection regime, age, and their interaction as fixed effects and biological replicates nested within the selection regime as random effects. Multivariate analysis was carried out in PAST version 3.1 [40]. Differences in daily fecundity as a function of age and longevity selection were evaluated by repeated measures ANOVA (with a univariate split plot approach) to correct for repeated measurements of the same sample throughout the experiment. Analysis was performed separately on daily fecundity over the 17 days of the experiment, as well as on measurements corresponding to 3, 9, 14, and 19 days of age. The impact of longevity selection and aging on locomotor activity performance was assessed by nested ANOVA on square root transformed data to meet the assumptions of the normality and homogeneity of variances. In all significant cases, multiple pair-wise comparisons were conducted using Tukey’s post-hoc test. To elucidate the pattern of variation in the NLFA profile in response to longevity selection and aging, the log-transformed proportions of NLFAs (corrected for block effects) and related (un)saturation indices (ΣSFAs, ΣMUFAs, ACL, PI, and TFAMEs) were subjected to principal component analysis (PCA), ANOVA on PCs scores and, Pearson's correlation coefficient between PCs eigenvector scores and each individual fatty acid. Linear regression analysis was implemented to test for possible correlations between fitness traits (fecundity and locomotor activity) and the proportional amount of each individual NLFA. To achieve equal sample sizes, the number of fecundity and locomotor activity samples (30 samples per replicate) was reduced to three samples per replicate using the average value of each of the 10 random samples as one data point. Given the correlation among hypotheses and variables, correction for multiple comparisons was not applicable [41].

## Results

### Fecundity

**Fig 1:**
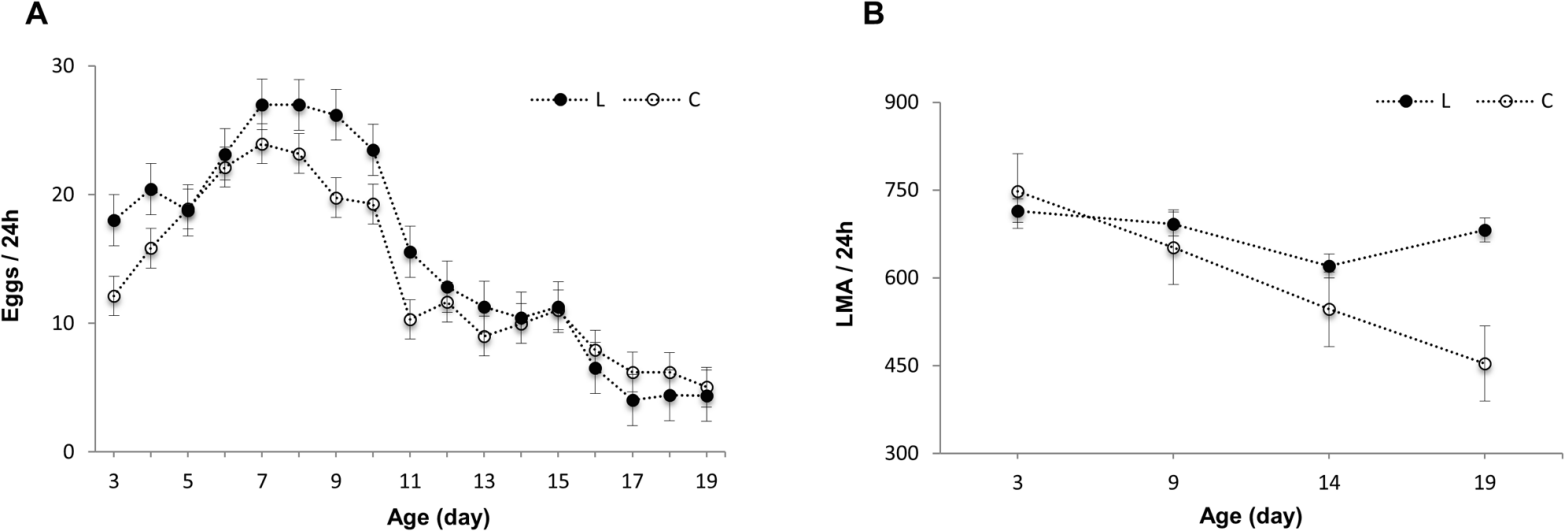
Fecundity (A) and locomotor activity (B) of the longevity-selected (black circle) and non-selected (white circle) control lines. Daily fecundity is expressed as the average number of eggs laid in 24 hours. Repeated-measures ANOVA showed that the 3- and 9-day-old L females were 48% (*P* = 0.02) and 32% (*P* = 0.01) more fecund, respectively, than their corresponding C lines. Levels of locomotor activity are represented as the average sum of movements in 24 hours. Tukey’s post-hoc comparisons showed a major difference between the locomotor activity of the L and C lines at 14 (*P* = 0.03) and 19 (*P* < 0.0001) days of age. Error bars indicate standard error of the mean.

The average daily fecundity curve (Fig 1A) in response to longevity selection and aging showed a consistent pattern between the L and C lines, with an increase in the average daily egg production up to day 7 along and gradual reduction thereafter. The number of eggs laid during the 17 days of the experiment (day 3 to 19) by L and C females was 264.39 ± 9.7 and 236.26 ± 9.9, respectively. Repeated-measures ANOVA indicated a strong significant effect of age (F_(3, 2984)_ = 995.80, *P* < 0.0001) and its interaction with selection regimes (F_(3,2984)_ = 28.69, *P* < 0.0001) on daily fecundity. The comparison of daily fecundity at 3, 9, 14, and 19 days of age in L and C lines indicated a significant effect of age (F_(3, 703)_ = 125.89, *P* < 0.0001), selection (F_(1, 703)_ = 8.31, *P* = 0.04) and their interaction (F_(3, 703)_ = 7.03, *P* = 0.0001) on female fecundity. No significant difference was observed in the daily fecundity of 3- and 14-day-old non-selected females (C_3_: 12.11 ± 0.9; C_14_: 10.06 ± 1, *P* = 0.4). A major difference between the L and C lines was observed in the first two age groups; the 3- and 9-day-old L females were 48% (*P* = 0.02) and 32% (*P* = 0.01) more fecund, respectively, than their corresponding C lines. Within each selection regime, females tended to lay more eggs early in life so that during the first 7 days (from day 3 to 9), L lines were significantly more fecund than C lines (L: 160.36 ± 4.54; C: 138.75 ± 4.63; F_(1,172)_ = 11.09, *P* = 0.02).

### Locomotor activity

The nested ANOVA revealed a considerable age effect on the pattern of locomotor activity (F_(3, 713)_ = 7.46, *P* < 0.0001), but the effect of longevity selection was not significant (F_(1, 713)_ = 0.84, *P* = 0.41). The significant interaction between age and selection regime (F_(3, 713)_ = 3.40, *P* = 0.02) indicated a different pattern of locomotor activity within each selection regime, with the level of activity in the L lines remaining approximately constant across the age groups (F_(3, 356)_ = 2.01, *P* = 0.11), but the C lines exhibited a decline in locomotor activity with advancing age (F_(3, 356)_ = 7, *P* = 0.0001; Fig 1B). Tukey’s post-hoc comparisons showed a major difference between the locomotor activity of the L and C lines at 14 (*P* = 0.03) and 19 (*P* < 0.0001) days of age.

### Storage lipid characterization

The storage lipid extract from intact female L and C flies contained eight major fatty acids with acyl chain lengths ranging between 12 and 18 C atoms. Among the detected fatty acids, myristic (C_14:0_), palmitic (C_16:0_), palmitoleic (C_16:1_), and oleic (C_18:1_) acid accounted for more than 88% of the total extract, whereas the abundance of the remnant fatty acids, containing lauric (C_12:0_), myristoleic (C_14:1_), stearic (C_18:0_) and linoleic (C_18:2_) acid, varied from 0.3 to 8% (Table 1).

**Table 1:** Mean proportion of neutral lipid fatty acids.

	Longevity-selected line				Control line			
	3 (Day)	9 (Day)	14 (Day)	19 (Day)	3 (Day)	9 (Day)	14 (Day)	19 (Day)
	Mean (± s.e.)	Mean (± s.e.)	Mean (± s.e.)	Mean (± s.e.)	Mean (± s.e.)	Mean (± s.e.)	Mean (± s.e.)	Mean (± s.e.)
**Saturated**								
C12:0	5.15 (0.35)	8.00 (0.37)	9.37 (0.39)	9.52 (0.41)	4.80 (0.28)	6.36 (0.032)	6.89 (0.43)	7.10 (0.41)
C14:0	31.96 (0.23)	35.68 (0.15)	36.05 (0.17)	35.62 (0.48)	30.17 (0.35)	33.37 (0.18)	33.01 (0.24)	32.33 (0.34)
C16:0	18.79 (0.39)	14.80 (0.41)	13.94 (0.44)	13.57 (0.37)	20.48 (0.43)	18.58 (0.37)	18.58 (0.42)	17.47 (0.35)
C18:0	0.71 (0.03)	0.37 (0.01)	0.33 (0.01)	0.31 (0.02)	0.82 (0.02)	0.44 (0.01)	0.41 (0.02)	0.37 (0.02)
**Monounsaturated**								
C14:1 (ώ 5)	1.60 (0.05)	2.15 (0.09)	2.18 (0.08)	2.07 (0.10)	1.38 (0.05)	1.67 (0.06)	1.57 (0.07)	1.59 (0.07)
C16:1 (ώ 7)	25.47 (0.38)	28.14 (0.38)	28.37 (0.41)	29.26 (0.53)	24.91 (0.24)	27.20 (0.49)	27.52 (0.51)	29.18 (0.51)
C18:1 (ώ 9)	14.87 (0.13)	10.02 (0.15)	9.03 (0.15)	8.91 (0.34)	15.91 (0.25)	11.61 (0.21)	11.29 (0.18)	11.13 (0.27)
**Polyunsaturated**								
C18:2 (ώ 6)	1.45 (0.03)	0.83 (0.02)	0.73 (0.02)	0.74 (0.03)	1.53 (0.05)	0.77 (0.03)	0.74 (0.03)	0.84 (0.06)
**(Un)saturation indices**								
ΣSFAs	56.61 (0.47)	58.86 (0.37)	59.68 (0.42)	59.02 (0.60)	56.27 (0.5)	58.75 (0.76)	58.89 (0.74)	57.27 (0.81)
ΣMUFAs	41.94 (0.47)	40.31 (0.38)	39.59 (0.41)	40.24 (0.59)	42.19 (0.45)	40.49 (0.73)	40.38 (0.72)	41.89 (0.79)
U/S	0.77 (0.01)	0.70 (0.01)	0.68 (0.01)	0.70 (0.02)	0.78 (0.02)	0.70 (0.02)	0.70 (0.02)	0.75 (0.02)
ACL	15.46 (0.01)	15.15 (0.02)	15.06 (0.02)	15.06 (0.03)	15.54 (0.02)	15.30 (0.02)	15.28 (0.02)	15.28 (0.03)
PI	2.49 (0.04)	1.84 (0.02)	1.72 (0.02)	1.74 (0.04)	2.59 (0.06)	1.78 (0.05)	1.75 (0.04)	1.89 (0.07)
**TFAMEs (mg/mg DW)**								
	0.06 (0.01)	0.08 (0.01)	0.10 (0.01)	0.09 (0.01)	0.06 (0.01)	0.10 (0.01)	0.11 (0.01)	0.09 (0.01)

Mean proportion is given on a mass basis, ± s.e. The mean proportion of each individual fatty acid was calculated as a percentage of the total amount of fatty acid methyl esters (TFAMEs). (Un)saturation indices are as follows: ∑SFAs = sum of saturated fatty acids, ∑MUFAs = sum of monounsaturated fatty acids, U/S = ratio of unsaturated to saturated fatty acids, ACL = average acyl chain length and PI = peroxidation index (see the materials and methods section for details).

**Fig 2:**
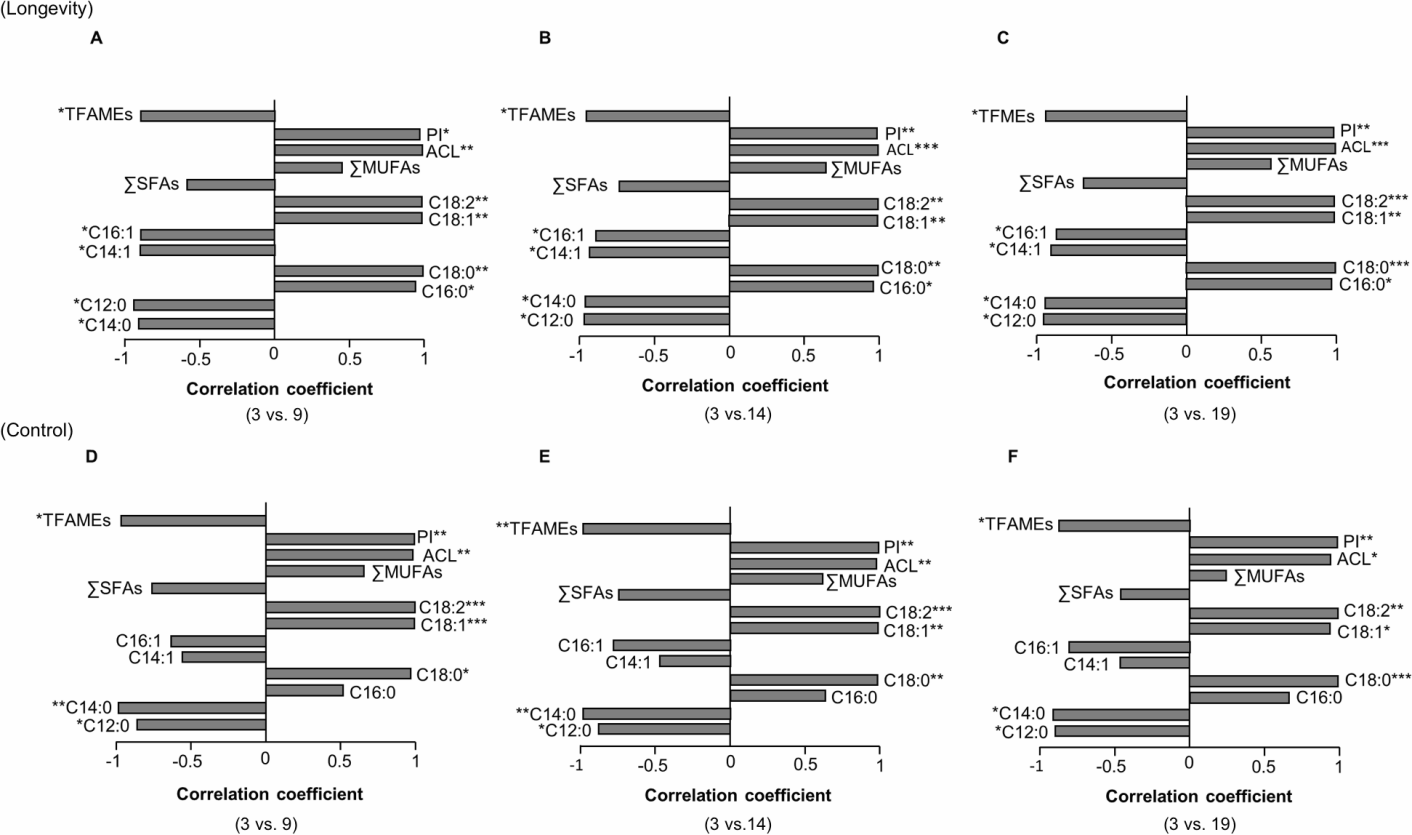
PCA loading plots of the age-associated effects on patterns of variation in neutral lipid fatty acids and related (un)saturation indices. Longevity-selected (A-C) and control lines (D-F) are shown separately. In both selection regimes, the fatty acid profiles of individuals at 3 days of age are significantly different from the profiles of other age groups (3 vs. 9: A, D; 3 vs. 14: B, E; 3 vs. 19: C, F). *P < 0.05, **P < 0.001, ***P < 0.0001.

In all PCAs, the first principal component (PC1) explained between 78% and 98% of the total variance among the 13 variables. In the assessment of age-related effects on the NLFA profile and (un)saturation indices within each selection regime, 3-day-old samples were significantly separated from the other age groups (9, 14 and 19 day old) along the PC1 axis, which was responsible for 88% to 95% of the total variance among selected variables. No significant difference was observed between the other age groups for any selection regime (Table 2). The differences between 3-day-old L individuals from the subsequent age groups depended on the significantly higher proportions (positive loading along the PC1 axis) of palmitic (C_16:0_), stearic (C_18:0_), oleic (C_18:1_), and linoleic (C_18:2_) acid and lower proportions (negative loadings along the PC1 axis) of lauric (C_12:0_), myristic (C_14:0_), myristoleic (C_14:1_) and palmitoleic (C_16:1_) acid (all *P* values < 0.05; Fig 2) in their storage fat profiles. The age-associated variations in the fatty acid profiles of the C lines were similar to the L lines except for insignificant correlations between myristoleic (C_14:1_), palmitic (C_16:0_), and palmitoleic (C_16:1_) acid and the PC1 scores (Fig 2D–2F). In both L and C lines, significant positive loading of the ACL and PI with PC1 axis indicated the presence of a higher proportion of long chain unsaturated fatty acids, and negative loading of the TFAMEs indicated a lower amount of storage lipid in the fat cells of flies in the early adult stage of life.

**Table 2:** Pair-wise comparison between ages.

		Longevity-selected line			Control line		
		3 (Day)	9 (Day)	14 (Day)	3 (Day)	9 (Day)	14 (Day)
9 (Day)	PC1 (%)	91.68			90.64		
	F ratio	107.8			98.22		
	*P*	**0.0005**			**0.0006**		
14 (Day)	PC1 (%)	95.57	55.25		92.62	78.33	
	F ratio	152.9	4.20		209.3	0.27	
	*P*	**0.0002**	0.11		**0.0001**	0.63	
19 (Day)	PC1 (%)	95.09	68.06	71.57	88.49	63.92	63.36
	F ratio	50.83	2.28	0.006	31.34	0.19	0.79
	*P*	**0.002**	0.21	0.94	**0.005**	0.69	0.43

The percentage of variation between variables is explained by the PC1 vector in a paired comparison between different age groups separately for longevity-selected and control lines. PC1 scores were subjected to ANOVA to find significant differences between age groups. Significant *P*-values (*P* < 0.05) are shown in bold.

**Fig 3:**
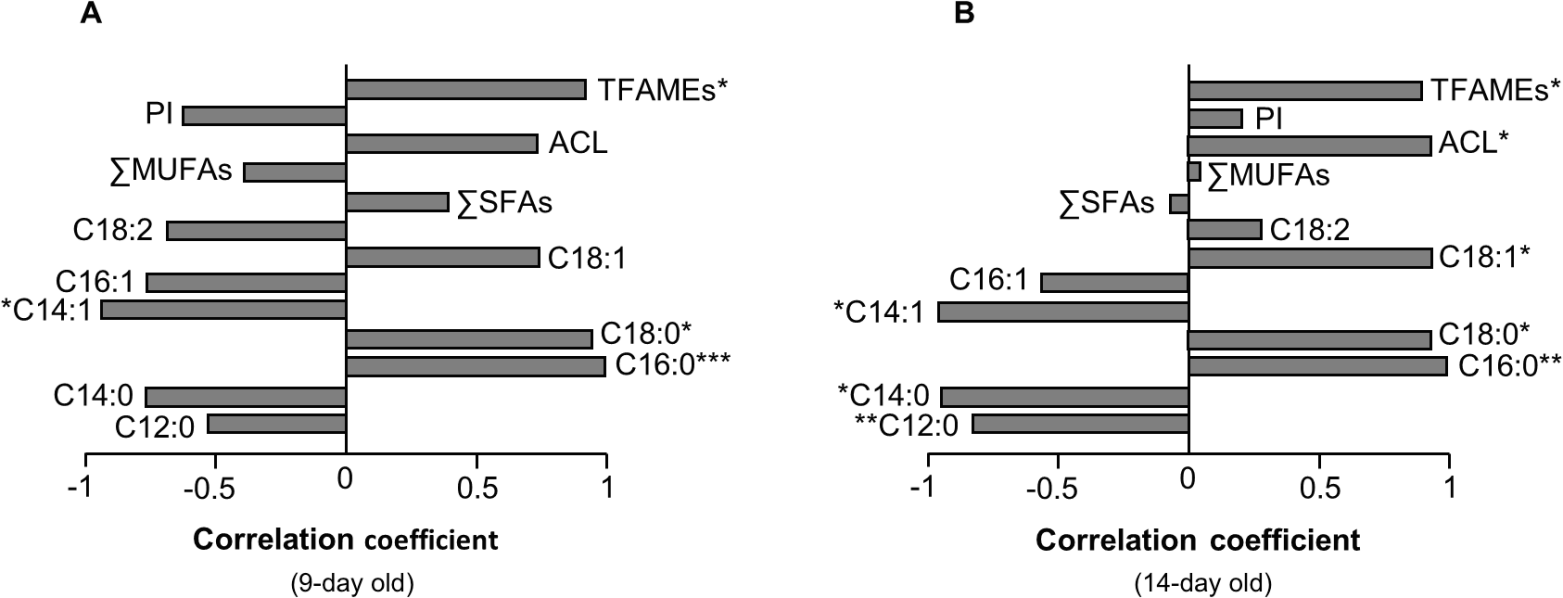
PCA loading plots assessing the effect of longevity selection on storage lipid composition. The fatty acid profiles of longevity-selected individuals were significantly different from control lines at 9 (*P* = 0.02; A) and 14 (*P* = 0.007; B) days of age. The loading plots represent the pattern of longevity selection-dependent alterations in eight neutral lipid fatty acid profiles and related (un)saturation indices along the PC1 axis. *P < 0.05, **P < 0.001, ***P < 0.0001.

PCA revealed a clear separation of 9- and 14-day-old L individuals from the corresponding C lines along the PC1 axis, which explained 68.76% (F_(1, 4)_ = 13.48, *P* = 0.02) and 82.40% (F_(1, 4)_ = 25.96, *P* = 0.007) of the total variation among variables, respectively. In both age groups, the lowest PC1 scores in loading plots belonged to the longevity selection regime. Based on the loading pattern of the NLFAs and subsequent (un)saturation indices along the PC1 axis as a function of selection (Fig 3), the proportion of fatty acids with ≥16 C atoms (e.g., C_16:0_ and C_18:0_) was higher and fatty acids with ≤14 C atoms (e.g., C_14:1_) was lower in C lines compared to L lines. Therefore, the ACL in 14-day-old C individuals was significantly longer than in the corresponding L lines (Fig 3B). In both age groups, the significant positive loading of TFAMEs along PC1 showed greater fat storage in the reserves of C lines compared to L lines (Fig 3).

### Correlations

Linear regression analysis revealed different correlations between the proportional amount of NLFAs and age-specific fecundity, as well as locomotor activity (Table 3). Within each selection regime, significant correlations varied across the age groups; the fecundity of 3-day-old L females was positively related to ΣSFAs (*P* = 0.02, R^2^ = 58.27%) and negatively related to ΣMUFAs (*P* = 0.01, R^2^ = 60.40%), but at older ages (14 and 19 days of age) these correlations were reversed (Table 3). None of the NLFAs and related (un)saturation indices correlated with the fecundity of non-selected lines, except 9- and 14-day-old samples. In 14-day-old C lines, ΣSFAs positively (*P* = 0.001, R^2^ = 79.83%) and ΣMUFAs negatively (*P* = 0.001, R^2^ = 80.09%) correlated with fecundity. In both selection regimes, the average egg production of 9-day-old individuals was strongly associated with the proportion of lauric acid (C_12:0_; L: *P* = 0.004, R^2^ = 72.22%; *P* = 0.04, R^2^ = 46.22%).

**Table 3:** Linear regression analysis.

		Fecundity						Locomotor activity					
		Longevity			Control			Longevity			Control		
	Age (day)	*R* ^*2*^ (%)	*P*	*ß*	*R* ^*2*^ (%)	*P*	*ß*	*R* ^*2*^ (%)	*P*	*ß*	*R* ^*2*^ (%)	*P*	*ß*
**C12:0**	3	2.86	0.66	1.18	0.38	0.87	-0.25	34.86	0.09	-91.98	5.21	0.55	33.84
	9	72.22	**0.004**	2.65	46.22	**0.04**	2.86	48.78	**0.04**	-128.19	0.11	0.93	-4.45
	14	0.03	0.96	-0.06	42.58	0.06	1.97	0.72	0.83	11.21	3.87	0.61	23.59
	19	22.83	0.19	-0.95	0.04	0.96	-0.03	11.91	0.36	43.29	0.34	0.88	-4.80
**C14:0**	3	1.96	0.72	1.49	0.12	0.93	-0.11	39.84	0.07	149.93	11.34	0.38	-40.56
	9	28.57	0.14	-4.03	9.13	0.43	2.30	27.04	0.15	230.88	0.16	0.92	-9.65
	14	1.43	0.76	0.88	4.38	0.59	1.16	2.01	0.72	-43.20	5.99	0.53	54.07
	19	12.54	0.35	-0.60	18.07	0.25	0.75	5.22	0.55	24.24	4.85	0.57	22.28
**C16:0**	3	38.48	0.07	3.87	3.10	0.65	-0.47	64.10	**0.01**	111.68	0.65	0.84	-7.88
	9	27.19	0.15	-1.46	3.90	0.61	0.72	71.41	**0.004**	139.61	12.23	0.36	-40.90
	14	41.93	0.06	-1.86	59.55	**0.01**	2.43	18.51	0.25	51.16	27.54	0.15	65.93
	19	6.66	0.50	-0.57	12.19	0.36	-0.60	22.59	0.20	65.65	35.52	0.09	-58.31
**C18:0**	3	61.99	**0.01**	61.74	40.74	0.06	-38.58	34.79	0.09	1034.75	41.71	0.06	1430.18
	9	0.79	0.82	-8.31	1.23	0.78	-23.99	8.38	0.45	1594.47	0.47	0.86	-476.53
	14	24.00	0.18	-42.67	20.14	0.23	38.94	5.35	0.55	832.21	44.32	0.05	2300.49
	19	0.96	0.80	3.87	39.19	0.07	17.71	3.57	0.63	-469.17	55.88	**0.02**	1208.18
**C14:1**	3	71.22	**0.004**	-43.76	5.02	0.56	5.08	13.62	0.33	-428.01	1.10	0.79	-87.25
	9	0.38	0.88	0.80	2.47	0.69	-3.41	12.48	0.35	-271.72	1.27	0.77	78.42
	14	42.44	0.06	10.18	57.59	**0.02**	-14.03	6.05	0.52	-158.79	50.17	**0.03**	-521.29
	19	0.46	0.86	-0.58	2.60	0.68	-1.30	1.32	0.77	-61.47	0.73	0.83	-39.47
**C16:1**	3	64.46	**0.01**	-5.20	14.15	0.32	1.79	33.57	0.10	-83.96	0.10	0.94	5.38
	9	2.68	0.67	0.50	15.81	0.29	-1.10	42.67	0.06	-116.68	10.37	0.40	28.47
	14	53.87	**0.02**	2.26	77.86	**0.002**	-2.26	43.96	0.05	-84.15	16.81	0.27	-41.76
	19	52.59	**0.03**	1.11	0.24	0.90	0.06	59.13	**0.02**	-73.86	6.86	0.50	17.56
**C18:1**	3	2.70	0.67	-3.18	0.23	0.90	0.22	5.81	0.53	-104.28	5.02	0.56	38.20
	9	28.51	0.14	-4.17	35.32	0.09	-3.79	50.52	**0.03**	326.60	1.60	0.75	-25.82
	14	23.56	0.19	-4.08	60.32	**0.01**	-5.62	30.72	0.12	192.31	38.28	0.08	-178.19
	19	4.83	0.57	0.53	9.29	0.43	-0.67	0.02	0.97	2.19	1.44	0.76	-14.94
**C18:2**	3	1.68	0.74	9.55	0.44	0.87	1.39	1.27	0.77	-186.01	11.00	0.38	254.72
	9	9.17	0.43	-17.71	21.95	0.20	-21.37	1.58	0.75	432.28	10.67	0.39	478.09
	14	4.56	0.58	-17.16	26.49	0.16	-24.81	25.42	0.17	1672.73	0.13	0.93	70.42
	19	2.73	0.67	4.35	45.12	0.05	6.16	5.66	0.54	-394.15	53.96	**0.02**	384.43
**∑SFAs**	3	58.27	**0.02**	3.97	5.61	0.54	-0.55	32.99	0.11	66.74	2.31	0.70	-12.87
	9	0.22	0.90	0.15	20.67	0.22	0.81	21.79	0.21	86.03	3.80	0.62	-11.15
	14	44.81	0.05	-2.04	79.83	**0.001**	1.58	23.46	0.19	60.84	25.23	0.17	35.45
	19	57.85	**0.02**	-1.03	0.11	0.93	0.02	49.11	**0.04**	59.39	3.04	0.65	-7.31
**∑MUFAs**	3	60.40	**0.01**	-4.06	6.36	0.51	0.64	32.47	0.11	-66.61	1.61	0.74	11.81
	9	0.09	0.94	-0.09	20.57	0.22	-0.84	21.74	0.21	-84.63	3.58	0.63	11.26
	14	46.27	**0.04**	2.08	80.09	**0.001**	-1.62	25.55	0.17	-63.75	26.56	0.16	-37.22
	19	58.98	**0.02**	1.06	0.79	0.82	-0.07	49.44	**0.03**	-60.84	1.40	0.76	5.09
**ACL**	3	2.42	0.69	-28.72	0.14	0.92	2.06	16.72	0.27	1688.71	0.57	0.85	152.50
	9	68.05	**0.01**	-61.47	46.73	**0.04**	-50.81	56.07	**0.02**	3284.93	0.00	0.99	9.61
	14	1.95	0.72	-8.68	43.41	0.05	-39.99	0.50	0.86	182.15	6.71	0.50	-626.26
	19	23.83	0.18	14.96	1.12	0.79	-2.36	8.50	0.45	-561.49	0.01	0.98	10.83
**TFAMEs**	3	2.94	0.66	-76.40	0.07	0.95	5.38	2.62	0.68	-1613.86	6.37	0.51	-1939.93
	9	11.02	0.38	-43.41	9.75	0.41	-61.92	5.34	0.55	1779.64	5.02	0.56	-1426.02
	14	4.65	0.58	-49.04	2.09	0.71	-37.59	0.91	0.81	-896.28	1.63	0.74	-1319.75
	19	45.37	0.05	-87.18	23.60	0.18	-29.81	19.87	0.23	3625.06	55.45	**0.02**	-2609.30

Linear regression analysis between each individual fatty acid along with (un)saturation indices and age-specific fecundity as well as locomotor activity of the longevity selected and control lines separately. The regression slope is indicated by *ß*. Significant correlations (*P* < 0.05) are shown in bold.

The linear regression of the average locomotor activity and the proportional amount of NLFAs indicated that L lines were more dependent than C lines on storage lipid to meet their energy requirements during active periods (Table 3). In the L lines, long chain fatty acids, such as palmitic (C_16:0_) and oleic (C_18:1_) acid and ΣSFAs provided the highest positive correlations with the average locomotor activity of young (C_16:0(3-day old)_: *P* = 0.01, R^2^ = 64.10%; C_18:1(9-day old)_: *P* = 0.03, R^2^ = 50.52%) and aged (ΣSFAs_(19-day old)_: *P* = 0.04, R^2^ = 49.11%) individuals. Interestingly, in older C flies (19 days of age), a negative correlation was observed between the TFAMEs and locomotor activity (*P* = 0.02, R^2^ = 55.45%).

## Discussion

Remodeling of NLFA composition in response to age, sex, development stage, and environmental condition plays a pivotal role in the maintenance of energy homeostasis with negligible variation in the fat content [23]. The impact of fatty acid composition on the physiochemical properties of storage lipids relies on metabolic discrimination between fatty acids as a function of their molecular structure. In the present study age-dependent modulation of the storage lipid composition and its potential to promote longevity is discussed with a critical reevaluation of the qualitative trade-offs between fecundity, locomotor activity and lifespan.

### Costs and benefits of fecundity

A negative correlation between fecundity and lifespan due to the direct and indirect costs of reproduction, such as energy allocation to mating and egg production, sexual harassment of females by males, and risk of oxidation due to elevation in oxidative metabolism, is a prevalent hypothesis [42]. Various methods employed to slow down the process of aging and prolong the lifespan of both *C*. *elegans* and *Drosophila* indicate that the inverse correlation between fecundity and longevity can be uncoupled under certain circumstances [43–45]. For example, lifespan extension via dietary restriction is usually accompanied by a reduction in fecundity [11,46], whereas Grandison et al. [12] found that dietary-restricted medium supplemented with methionine increased the lifespan of adult flies without any trade-off with fecundity. Uncoupling the trade-off between longevity and reproduction does not abolish the energetic costs of survival, because they may affect other aspects of fitness [47].

In this study, over the first 7 days, L females out-performed the C individuals (Fig 1). In a recent study of the lines used for the present experiment, the lifetime fecundity of the L lines was higher than that of the C lines [28]. These observations indicate that a reduction in fecundity (based on the number of eggs) does not appear to be essential for extending the lifespan of longevity-selected lines. Competition for limited energy resources has been considered as the major physiological reason associated with the survival costs of reproduction [47]. Thus, the higher egg-laying rate of the L lines and consequently higher energy investment in reproduction may be compensated by alterations in other fitness components, such as egg size and egg survival (fertility). The absence of a difference in the mean daily metabolic rate between the L and C lines based on the rate of CO_2_ production [28] supports the presence of such compensatory factors. Metabolic reprogramming and the diversion of energy from somatic maintenance to reproduction through improved mitochondrial function may be another strategy of providing sufficient energy resources for the longevity-selected lines to be both fecund and longevous [48], though the reason behind such energy use in response to longevity selection remains unknown.

The costs associated with fecundity arise from embryogenesis, when the lipid content of insect oocytes increases several fold during a short period of time to obtain adequate fuel for the developing embryo [17,49]. Lipids accumulated in the oocytes are transported from the fat body to the ovaries via lipoproteins in the form of diacylglycerol [30]. The accumulation of lipids in oocytes is associated with a remarkable reduction in the amount of fat storage [50]. This feature triggers the secretion and production of body fat-derived hormones such as adipokines improving the lipid metabolism and immune responses [9,51]. The secretion of adipokinetic hormone relies on endocrine signals emanating from germline stem cells. These signals increase lifespan by modulating lipid hydrolysis [52]. Due to this feature, germline ablation in *C*. *elegans* and *Drosophila* is accompanied by enhanced lipid hydrolysis [7,53].

Thus, fecundity cannot only be considered an energetically costly process, but may also be partly beneficial by preventing fat accumulation during adulthood. In many species reduced reproduction with advancing age is associated with increased body fat mass [54]. These observations may indicate a positive contribution of fecundity on lifespan. In the present study, the higher rate of egg production in the L vs. C lines at 9 days of age along with their significant lower amount of TFAMEs support the link between fecundity, body fat mass, and lifespan (Figs 1A and 3A).

The age-related fecundity patterns in the L and C lines were similar to the patterns in a previous study on lifetime fecundity in these lines, with a rapid increase in daily fecundity at early ages and a gradual reduction thereafter [28]. However, the average daily egg production and the day at which females reached peak fecundity were different between the two studies. These inconsistencies may arise from differences in the protocols used to assay the fecundity; the presence of males in the previous study may have induced female egg production [55].

### Age-related pattern of locomotor activity

Aging is characterized by a progressive decline in major physiological and behavioral processes due to an accumulation of molecular damage [56]. Evaluating the regulation of age-associated genes in *D*. *melanogaster* showed that muscles are sensitive to senescence [57]. Here, reduction in locomotor activity in the C lines supported the impact of aging on flight muscles (Fig 1B).

Contradictory observations from the assessment of age-associated alterations in physical activity may arise from different methods and protocols used to evaluate locomotor activity. In the majority of previous assays, flight activity has been considered a proxy of locomotor activity [58–60], but in the present experiment locomotor activity was restricted to physical activity under housing conditions. Given the higher energy expenditure during flight activity compared to walking [61,62], one can claim that assessing the locomotor activity by DAMs minimizes differences in energy requirements between the L and C lines. The higher activity (>55%; *P* < 0.0001) of the L lines compared to the C lines on day 19 supports the adequacy of this estimate. This feature may indicate better health of the L females compared to the C females at 19 days of age due to their lower body fat mass and lower risk of fat-associated diseases. Nevertheless, oxygen consumption as a function of physical activity may increase the risk of oxidation by reactive oxygen species [63]. Resistance to oxidation may arise from better functioning of the antioxidant defense system and mitochondria, attenuating the activity-induced oxidative stress [64].

Differences in the patterns of locomotor activity for the L and C lines may represent the variation in energy consumption, and the physical activity of the L lines may not be impaired by age in the same time frame as the C lines. Re-allocation of energy from reproduction to somatic maintenance is one of the common strategies for increasing lifespan, along with an improvement in other fitness components [25,65,66]. However, such a trade-off was not observed in the present study.

### Fat storage, aging and longevity

Storage lipids play a fundamental role in the life cycle of holometabolous insects because of their high calorie content/unit weight and anhydrous form of storage in lipid droplets [17,67]. The type and level of fatty acids accumulated in the fat cells depend on several factors, such as nutrition, sex, and development stage. Here, eight fatty acids comprising four SFAs, three MUFAs, and one PUFA were identified within the storage lipid extract of the L and C lines at different ages (Table 1). Among the detected fatty acids, lauric (C_12:0_) and myristoleic (C_14:1_) acid were not present in the *Drosophila* standard medium, which indicates the de novo synthesis of these fatty acids. Kiyomoto and Keith [68] demonstrated that *Drosophila* larvae are able to synthesis MUFAs by direct desaturation of the existing saturated homolog.

The necessity of marginal biosynthesis of lauric (C_12:0_) and myristoleic (C_14:1_) acid in *D*. *melanogaster* may be due to their incorporation into egg production and embryo development (Table 3). Fast and efficient hydrolysis of fatty acids with 12 or fewer C atoms compared to long chain fatty acids [16] designates short chain fatty acids as excellent substrates for the developing embryo. However, further investigation of the fatty acid composition of eggs laid by females is needed to support the contribution of lauric (C_12:0_) and myristoleic (C_14:1_) acid to egg development.

Interestingly, comparison of the composition of cell membrane phospholipids in the L and C lines [39] and neutral lipid fatty acids suggests that *D*. *melanogaster* belongs to a group of insects capable of synthesizing PUFAs using desaturase enzymes. The presence of linolenic acid (C_18:3_) among the phospholipid components [39] probably results from desaturation of linoleic acid (C_18:2_), the only PUFA detected among the neutral lipids.

The separation of 3-day-old samples from the other age groups stemmed from variations in the proportion of long chain unsaturated fatty acids in the early adult stage of life. This difference was achieved by high incorporation of palmitic (C_16:0_), stearic (C_18:0_), oleic (C_18:1_), and linoleic (C_18:2_) acid in the composition of storage lipids of 3-day-old flies (Fig 2). This finding is partly in accordance with previous work by Green and Geer [21] on age-related variations in the fatty acid composition of *D*. *melanogaster*. The considerable amount of unsaturated fatty acids in 3-day-old adult flies may be due to a larval preference to accumulate more unsaturated fatty acids than saturated fatty acids in fat cells [69], which are present within the abdomen of adult flies until 3–4 days after eclosion [70]. In addition, the concentration of the MUFAs was reported to increase during the pupal stage concomitant with the development of imaginal discs [21].

Chronological variation in the fatty acid composition of neutral lipids shows the dynamic structure of the fat body. This variation may be derived from age-related changes in the contribution of storage lipids as a major source of energy in various phenotypic traits, such as flight, reproduction, and stress resistance [52,71,72]. Here, the correlation between neutral lipid fatty acids and fecundity, as well as locomotor activity, varied across age groups and between L and C lines (Table 3). Given that the lipid metabolism is a complex physiological mechanism, the higher metabolic dependence of L lines on fatty acids can be interpreted as an energy utilization strategy due to the high potential of lipids to contribute to catabolic pathways and to generate energy.

The molecular structure of fatty acids has a profound impact on their metabolism [16]. Therefore, different compositions of fatty acids form storage lipids with different physiochemical properties [73]. Based on this explanation, the considerable abundance of fatty acids with 14 or fewer C atoms in the composition of storage lipids of L flies may reduce the phase transition temperature and improve fluidity of storage lipids. This feature facilitates the lipolysis of storage lipids by lipases and prevents age-associated fat accumulation [74]. Conversion of 18C fatty acids into shorter chain components in *D*. *melanogaster* cultured in medium supplemented with stearic (C_18:0_) and oleic (C_18:1_) acid represents the preference of this species for fatty acids with shorter acyl chain tails [20].

The lipolysis of storage lipids relies on the activity of fat body lipases. Over-expression of these enzymes sometimes increases the lifespan of model organisms [6,52] For example, overexpression of the triglyceride lipase *lipl-4* increased the lifespan in germline-ablated *and* fertile individuals of *C*. *elegans* [7,73,75]. In further studies, the TOR signaling pathway was identified as the major regulator of lipase *lipl-4* [76]. In addition, the TOR signaling extends lifespan through inducing autophagy, which has been directly linked to aging and longevity [77,78].

Fat storage also contributes to the process of aging through fat-body-derived signaling factors (adipokines) that modulate lipid accumulation in the fat droplets, increase the rate of fatty acid oxidation, and improve insulin sensitivity [6,9]. A negative correlation between lifespan and body fat mass is demonstrated by the impact of calorie restriction on the extension of lifespan through a reduction in storage lipids [15,79,80]. The present results are consistent with this notion, as the amount of storage lipids was significantly lower in the L lines compared to the C lines for 9- and 14-day-old individuals. The lower amount of TFAMEs in 9-day old individuals of the L lines may arise from their higher rate of fecundity and the locomotor activity. These two factors may stimulate the lipolysis of storage lipids by enhancing the secretion of adipokines [81]. The influence of fat body on lifespan should not be considered a simple scenario because there are a lot of factors including the fat body composition and its distribution, which affect the rate of aging [73].

## Conclusion

This study evaluates the age-associated variation in fat storage composition and its impact on lifespan. The negative correlation between lifespan and body fat mass highlights the role of storage lipids in the aging process. This association relies on the importance of maintaining energy homeostasis on the survival and reproductive success of organisms [6,82]. Here, the lower amount of storage lipids in 9-day old females of the L lines may be a result of the higher fecundity and locomotor activity in the early adult stage, which may increase the rate of lipid metabolism via alterations in metabolic pathways. This conclusion requires further clarification of the fat body contribution in fecundity, as well as locomotor activity and the influence of these two factors on pathways involved in the process of aging.
